# Diagnostic potential of energy metabolism-related genes in heart failure with preserved ejection fraction

**DOI:** 10.3389/fendo.2023.1296547

**Published:** 2023-11-27

**Authors:** Qiling Gou, Qianqian Zhao, Mengya Dong, Lei Liang, Hongjun You

**Affiliations:** ^1^ Department of Cardiovascular Medicine, Shaanxi Provincial People’s Hospital, Xi’an, Shaanxi, China; ^2^ Department of Cardiopulmonary Rehabilitation, Xi’an International Medical Center Hospital-Rehabilitation Hospital, Xi’an, Shaanxi, China

**Keywords:** heart failure with preserved ejection fraction, energy metabolism-related genes, diagnostic biomarkers, machine learning, immunoscape, targeted drug prediction

## Abstract

**Background:**

Heart failure with preserved ejection fraction (HFpEF) is associated with changes in cardiac metabolism that affect energy supply in the heart. However, there is limited research on energy metabolism-related genes (EMRGs) in HFpEF.

**Methods:**

The HFpEF mouse dataset (GSE180065, containing heart tissues from 10 HFpEF and five control samples) was sourced from the Gene Expression Omnibus database. Gene expression profiles in HFpEF and control groups were compared to identify differentially expressed EMRGs (DE-EMRGs), and the diagnostic biomarkers with diagnostic value were screened using machine learning algorithms. Meanwhile, we constructed a biomarker-based nomogram model for its predictive power, and functionality of diagnostic biomarkers were conducted using single-gene gene set enrichment analysis, drug prediction, and regulatory network analysis. Additionally, consensus clustering analysis based on the expression of diagnostic biomarkers was utilized to identify differential HFpEF-related genes (HFpEF-RGs). Immune microenvironment analysis in HFpEF and subtypes were performed for analyzing correlations between immune cells and diagnostic biomarkers as well as HFpEF-RGs. Finally, qRT-PCR analysis on the HFpEF mouse model was used to validate the expression levels of diagnostic biomarkers.

**Results:**

We selected 5 biomarkers (Chrna2, Gnb3, Gng7, Ddit4l, and Prss55) that showed excellent diagnostic performance. The nomogram model we constructed demonstrated high predictive power. Single-gene gene set enrichment analysis revealed enrichment in aerobic respiration and energy derivation. Further, various miRNAs and TFs were predicted by Gng7, such as Gng7-mmu-miR-6921-5p, ETS1-Gng7. A lot of potential therapeutic targets were predicted as well. Consensus clustering identified two distinct subtypes of HFpEF. Functional enrichment analysis highlighted the involvement of DEGs-cluster in protein amino acid modification and so on. Additionally, we identified five HFpEF-RGs (Kcnt1, Acot1, Kcnc4, Scn3a, and Gpam). Immune analysis revealed correlations between Macrophage M2, T cell CD4+ Th1 and diagnostic biomarkers, as well as an association between Macrophage and HFpEF-RGs. We further validated the expression trends of the selected biomarkers through experimental validation.

**Conclusion:**

Our study identified 5 diagnostic biomarkers and provided insights into the prediction and treatment of HFpEF through drug predictions and network analysis. These findings contribute to a better understanding of HFpEF and may guide future research and therapy development.

## 1 Introduction

Heart failure with preserved ejection fraction (HFpEF) is a type of heart failure that occurs when the diastolic function of the heart is impaired. In HFpEF, the cardiac ejection fraction remains within the normal range, but the condition is characterized by ventricular underfilling, left atrial dilatation, and elevated left atrial pressure (1). The prevalence of HFpEF is believed to be increasing and is associated with factors such as age, gender, obesity, and hypertension (2). The diagnosis of HFpEF relies on clinical symptoms, electrocardiogram, cardiac ultrasound, and other examination methods, but there are no specific diagnostic biomarkers (3). Therefore, it is crucial to identify potential biomarkers that can aid in the accurate diagnosis of HFpEF.

Energy metabolism is essential for maintaining normal physiological functions in the body, including processes like glycolysis, the tricarboxylic acid cycle, and oxidative phosphorylation. The heart’s energy supply primarily relies on the metabolism of glucose, fatty acids, and lactate (4). Genes and proteins related to energy metabolism play critical roles in various human diseases, including cardiovascular disease (5), diabetes, and obesity (6). A deeper understanding of the molecular mechanisms underlying energy metabolism may provide insights into novel therapeutic approaches for HFpEF (7, 8).

To identify potential diagnostic biomarkers and therapeutic targets for HFpEF, this study conducted bioinformatics analysis using the mouse HFpEF transcriptome from the GEO database. Through screening and research, we identified five potential biomarkers and constructed a regulatory network based on energy metabolism-related genes. These biomarkers have the potential to serve as diagnostic biomarkers for HFpEF and may provide a foundation for discovering new therapeutic targets.

## 2 Materials and methods

### 2.1 Source of data

The GSE180065 dataset was sourced from the GEO database (https://www.ncbi.nlm.nih.gov/geo/query/acc.cgi?acc=GSE180065). The GSE180065 dataset (GPL24247) comprises RNA-seq data obtained from heart tissue samples of 10 HFpEF mice and five control mice. To construct the HFpEF mouse model in this publicly available dataset, wide-type male C57BL/6J mice were treated with a combination of high-fat diet and N[w]-nitro-l-arginine methyl ester (L-NAME) at a concentration of 0.5g/L for five weeks. The 325 energy metabolism-related genes (EMRGs) were downloaded from the NCBI and MsigDB databases.

### 2.2 Identification of DEGs

DEGs between the HFpEF and control groups were chosen by using the DESeq2 package (v 1.36.1) (9) in the GSE180065 dataset at P value < 0.05 and |log_2_FC| > 0.5. The results of the differential analysis were illustrated by volcano map plotted by the ggplot2 package (v 3.4.1) (10). Next, the DEGs were intersected with the EMRGs to obtain DE-EMRGs.

### 2.3 Machine learning screening and performance evaluation of biomarkers

Three machine learning models were constructed based on DE-EMRGs by least absolute shrinkage and selection operator (LASSO), random forest (RF) and Support Vector Machine-Recursive Feature Elimination (SVM-RFE) algorithms to screen feature genes separately. LASSO regression profiling was carried out using the glmnet package (version 4.1-6) (11) to obtain LASSO-feature genes. RF analysis was performed using the caret package (v 6.0-86) based on DE-EMRGs, and genes with top 10 importance scores were screened as RF signature genes. Next, SVM analysis was performed. Finally, the genes included in the portfolio with the highest accuracy rate and lowest error rate were selected as SVM-RFE-feature genes. The biomarkers were screened by overlapping LASSO-feature genes, RF-feature genes and SVM-RFE-feature genes.

Subsequently, ROC curves were plotted using the pROC package (v 1.17.0.1) (12) to assess the diagnostic value of the biomarkers. In addition, logistic regression models were constructed using the biomarkers as a whole and the model was evaluated using ROC curves. Immediately after, the nomogram was constructed and visualized via regplot (v 1.1) and rms package (v 4.1-1) (13). Next, the calibration curve was plotted to judge the model performance.

### 2.4 Single-gene GSEA analysis

In order to further explore the biomarkers related pathways and the functions they play, we performed a single-gene GSEA analysis. The single-gene GSEA analysis of biomarkers was carried out via clusterProfiler package (v 4.4.4) (14). The top 10 most significant results for each biomarker were visualized separately.

### 2.5 Construction of mRNA-drug interaction and TF-mRNA-miRNA networks

In order to find potential therapeutic small molecule drugs acting on biomarkers, we performed drug prediction. The drugs targeting the biomarkers (transformation into human genes) were predicted through the DrugBank database. A mRNA-drug network was constructed based on the predicted results. Then, NetworkAnalyst database was utilized to predict the targeting miRNAs and TFs of biomarkers. Lastly, the network was visualized using Cytoscape software (v 3.8.2) (15).

### 2.6 Consensus clustering analysis

The consensus clustering analysis was performed on the GSE180065 dataset utilizing the ConsensusClusterPlus package (v 1.60.0) (16) on the basis of biomarkers.

### 2.7 Screening and enrichment analysis of DEGs-cluster

DEGs-cluster between the subtypes were selected via the DESeq2 package (v 1.36.1) (9) with P < 0.05 and |log_2_FC| > 0.5. Gene Ontology (GO) enrichment analysis of DEGs-cluster was executed via clusterProfiler package (v 4.4.4) (14) (P value < 0.05) and org.Mm.eg.db package (v 3.12.0).

### 2.8 Screening for HFpEF-related genes

This part of the analysis was carried out in order to obtain further information on the genes associated with the development of HFpEF. Firstly, the DEGs was intersected with the DEGs-cluster to obtain the intersected genes. Then, the protein-protein interaction (PPI) network was created on the basis of intersected genes via the STRING database (https://cn.string-db.org/). In this study, the topology of the PPI network was analyzed using the plugin cytoHubba, and the top 5 genes under the MCC algorithm were selected as HFpEF-RGs for subsequent analysis.

### 2.9 Immune-infiltration analysis

To obtain correlations of subtypes and biomarkers with immune cells, we performed an immune infiltration analysis. The immune score and proportions of immune cell subtypes for each sample in the GSE180065 dataset were computed via the xcell algorithm of the immunedeconv package (v 2.0.4) (17). In the first step, the differences in abundance of each immune cell between HFpEF and the control groups (differential immune cells 1) were compared and the results were presented by box plots. Then, differences in the proportion of immune cells were analyzed between subtypes (differential immune cells 2). In addition, the correlation of biomarkers with differential immune cells1 and the association of HFpEF-RGs with differential immune cells2 were computed using the Spearman method.

### 2.10 A ‘two-hit’ mouse model of HFpEF and echocardiography

Male wild-type (WT) C57BL/6 mice weighing about 20 g at the age of 8 weeks were obtained from the Hu’nan Silaikejingda Experimental Animal Co., Ltd, China. All applicable international and national guidelines for the care and use of animals were followed. Mice were divided into two treatment groups and exposed to a combination of a high-fat diet (HFD) (60% kilocalories from fat) and Nω-nitro-L-arginine methyl ester (L-NAME) (0.5 g l−1 in drinking water) or a standard (chow) diet for 15 weeks (18). The concomitant metabolic stress (obesity and metabolic syndrome) and mechanical stress (hypertension induced by constitutive NO synthases suppression) in mice—elicited by the aforementioned ‘two-hit’—recapitulates the numerous systemic and cardiovascular features of HFpEF in humans. Transthoracic echocardiography was performed on mice at 15 weeks after treatment. A two-dimensional echocardiographic system (Philips iE33, Netherlands) was used to examine the cardiac function of the left ventricle by detecting and calculating the left ventricular systolic and diastolic indexes.

### 2.11 RNA isolation and quantitative real-time polymerase chain reaction

Eight pairs of frozen left ventricle tissue of mouse heart (8 HFpEF and 8 control samples) were collected. Afterwards, 16 samples were lysed with TRIzol reagent and total RNA was isolated following the manufacturer’s instructions. The concentration of RNA was measured with a NanoPhotometer N50. Afterwards, RNA was reverse transcribed into cDNA using the SureScript First strand cDNA synthesis kit (Servicebio, Wuhan, China). The qRT-PCR reaction consisted of 3 µL of reverse transcription product, 5 µL of 2xUniversal Blue SYBR Green qPCR Master Mix, and 1 µL each of forward and reverse primer. All primer sequence information were shown in **Table 1**. The GAPDH gene served as an internal control, and the relative expression of genes was determined using the 2^-ΔΔCT^ method (19). Graphpad Prism 5 was used to make the graph and calculate the p-value.

**Table 1:** Primer sequences of PCR.

Gene	Forward primer (5–3)	Reverse primer (5–3)
Chrna2	AACAATGCAGACGGGGAGTTT	GGGAAGAAAGTCACGTCGATG
Gnb3	ATACTCCAGGGGCCATTCCT	GGGGAAGGGGTCCATTCTTG
Gng7	GCTTTGCTATATCGAGCCTGC	CCCAGCACTGAGGTTCCAAT
Ddit41	TGGATAGGATCGTGTGTGATGC	CGTTCCAATCAGGGAGTACAGTT
Prss55	CTGCTACTTGTTGCCCACAC	GAGGCGAGGAGAGCAGGTAT
GAPDH	CCTTCCGTGTTCCTACCCC	GCCCAAGATGCCCTTCAGT

### 2.12 Statistical analysis

All bioinformatics analyses were carried out in R language. And then, the data of different groups were compared by rank sum test. It was a truism that *P* < 0.05 was in significant difference, where *P* < 0.05: *, *P* < 0.01: **, *P* < 0.001: ***, and *P* < 0.0001: ****.

## 3 Results

### 3.1 Screening of DE-EMRGs

**Figure 1:**
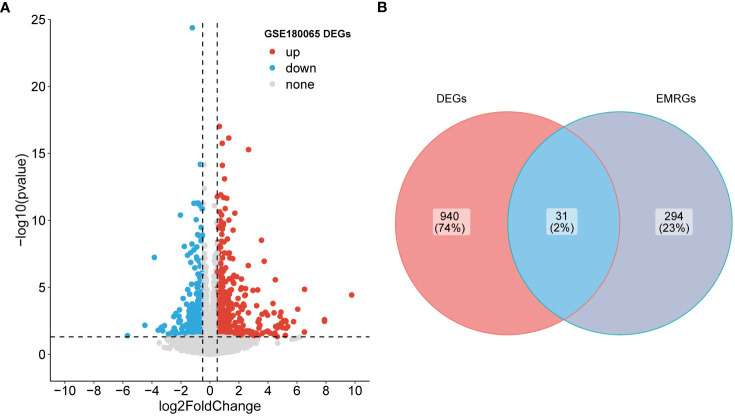
Identification of DE-EMRGs. **(A)** Volcano plot of DEGs. Red and blue dots indicate upregulated and down-regulated genes, respectively, and gray is the non-significant gene. **(B)** The intersection of DEGs with EMRGs. DE-EMRGs, differentially expressed energy metabolism-related genes; DEGs, differentially expressed genes; EMRGs, energy metabolism-related genes.

A total of 971 DEGs were identified through differential expression analysis based on the GSE180065 dataset (P value < 0.05 and |log_2_FC| > 0.5; **Supplementary Table 1**). Among them, 599 genes were significantly upregulated, and 372 genes were significantly downregulated (**Figure 1A**). To identify the EMRGs within these DEGs, an intersection analysis was performed. The results revealed 31 overlapping genes (**Figure 1B**), with 18 genes showing upregulation and 13 genes showing downregulation in expression (**Supplementary Table 2**). Hence, these genes were defined as DE-EMRGs.

### 3.2 Screening and performance evaluation of biomarkers

**Figure 2:**
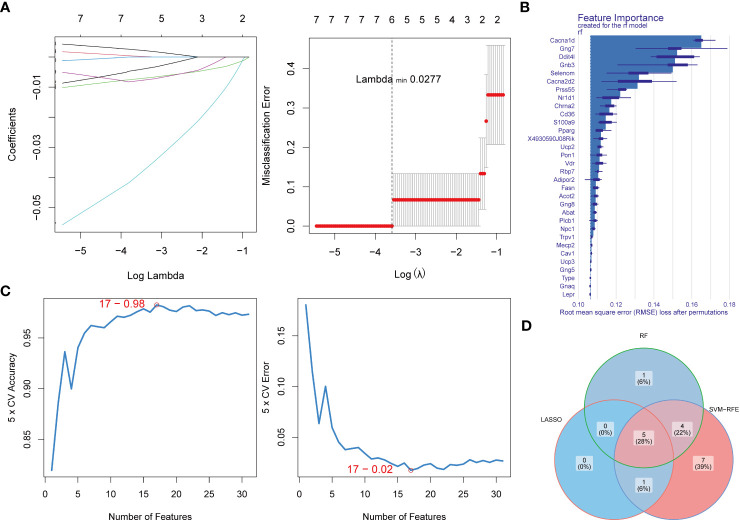
Identification of key DE-EMRGs using machine learning algorithms. **(A)** LASSO logistic regression algorithm used to screen key genes. **(B)** The importance of the variables was ranked by RMSE loss after permutations, with higher values indicating that the variable contributes greater to the accuracy of the model. **(C)** SVM-RFE algorithm to screen diagnostic biomarkers. **(D)** Venn diagram demonstrates the intersection of diagnostic biomarkers obtained by the three algorithms. DE-EMRGs, differentially expressed energy metabolism-related genes; LASSO, least absolute shrinkage and selection operator; RMSE, root mean square error; SVM-RFE, support vector machine-recursive feature elimination.

In order to obtain biomarkers, we filtered DE-EMRGs using 3 machine learning algorithms. A total of 6 LASSO-feature genes (Chrna2, Rbp7, Gnb3, Gng7, Ddit4l, and Prss55) were screened by LASSO regression analysis (**Figure 2A**). Subsequently, a total of 10 RF-feature genes (Cd36, Chrna2, Nr1d1, Prss55, Cacna2d2, Selenom, Gnb3, Ddit4l, Gng7, and Cacna1d) were obtained after RF analysis (**Figure 2B**). The accuracy and error rate were computed and found that the SVM model had the highest accuracy rate and lowest error rate when it contained 17 genes (**Figure 2C**). Therefore, these 17 genes were selected as SVM-RFE-feature genes (Acot2, Cacna1d, Cacna2d2, Rbp7, Gnb3, Ddit4l, Chrna2, Selenom, Gng7, Prss55, Pparg, Ucp2, Nr1d1, Vdr, Npc1, S100a9, and 4930590J08Rik) for further analysis. Hence, a total of 5 biomarkers (Chrna2, Gnb3, Gng7, Ddit4l, and Prss55) were screened by overlapping LASSO-feature genes, RF-feature genes and SVM-RFE-feature genes (**Figure 2D**).

**Figure 3:**
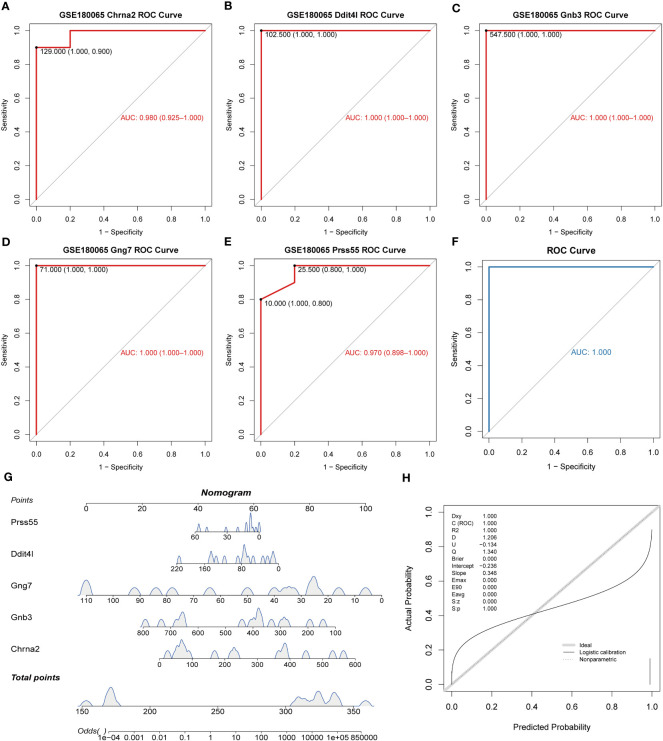
Diagnostic value of key DE-EMRGs in HFpEF. **(A–E)** The ROC curves of the key DE-EMRGs. **(F)** ROC curves of the diagnostic model in the GSE180065 dataset. **(G)** Nomogram for HFpEF samples. **(H)** Calibration curve to assess the predictive power of the nomogram. DE-EMRGs, differentially expressed energy metabolism-related genes; HFpEF, heart failure with preserved ejection fraction; ROC, receiver operating characteristic.

The ROC results revealed excellent diagnostic performance for both biomarkers and logistic regression models (**Figures 3A–F**). The nomogram on the basis of biomarkers was utilized to predict the risk of patients developing HFpEF (**Figure 3G**). The accuracy of the nomogram was relatively high, which was validated by the calibration curve (**Figure 3H**).

### 3.3 Single-gene GSEA analysis of biomarkers

**Figure 4:**
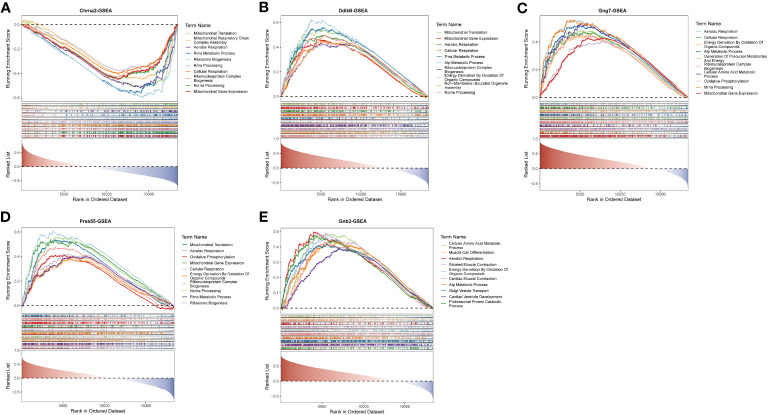
Single gene GSEA-GO for the 5 key DE-EMRGs. Enrichment in GO collection by Chrna2 **(A)**, Ddit4l **(B)**, Gng7 **(C)**, Prss55 **(D)**, and Gnb3 **(E)**. Each line represents one gene set with unique color. Gene sets were considered significant only when |NES| > 1, *P* < 0.05, and q < 0.05. Only several leading gene sets (Top 10) were displayed in the plot. GSEA, gene set enrichment analysis; GO, Gene Ontology; DE-EMRGs, differentially expressed energy metabolism-related genes; NES, normalized enrichment score.

In quick succession, single-gene GSEA was performed to explore the enriched regulatory pathways and molecular functions of each biomarker. Chrna2, Gng7, Ddit4l, and Prss55 were mainly enriched to GO terms such as aerobic respiration, ribonucleoprotein complex biogenesis, etc. (**Figures 4A–D**; **Supplementary Tables 3**-**6**). Gnb3 was mainly enriched to ribosome, energy derivation by oxidation of organic compounds, cellular amino acid metabolic process and so on (**Figure 4E**; **Supplementary Table 7**).

### 3.4 The TF-mRNA-miRNA and mRNA-drug networks of biomarkers

**Figure 5:**
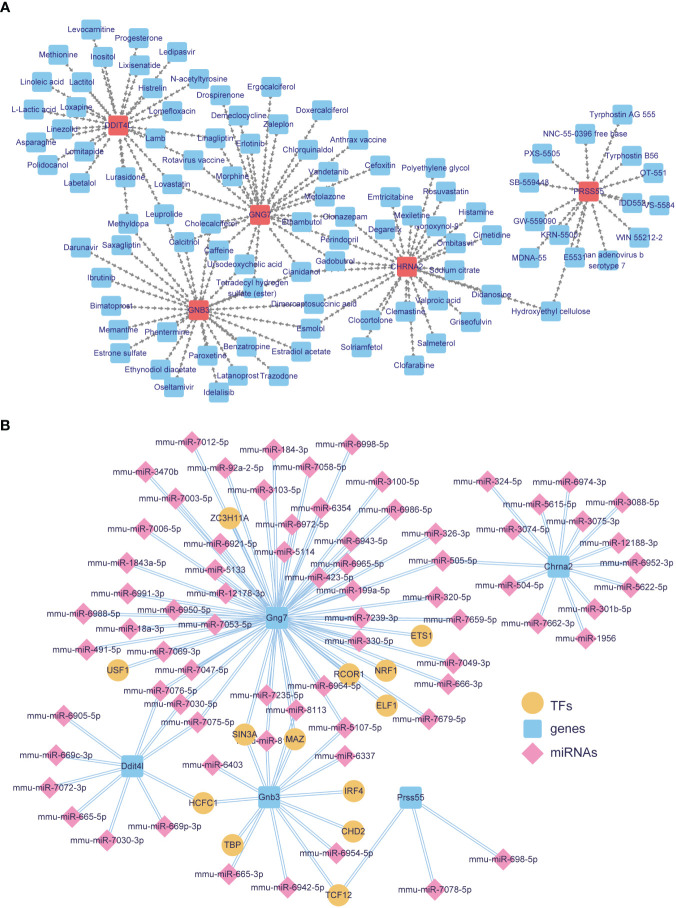
Targeted drugs and regulatory networks for key DE-EMRGs. **(A)** DrugBank-based drug-key DE-EMRGs interaction network. **(B)** Integrated miRNA-key DE-EMRGs and key DE-EMRGs-TFs interaction networks for the 5 biomarkers. Blue squares represent nine hub genes. Yellow circles represent TFs that have connectivity with biomarkers. Pink diamonds represent miRNAs associated with biomarkers. DE-EMRGs, differentially expressed energy metabolism-related genes; miRNA, microRNA; TFs, transcription factor.

Considering the targeting drugs and regulatory factors of these diagnostic biomarkers, we constructed the mRNA-drug and TF-mRNA-miRNA networks. Through DrugBank database, 5 biomarkers were found that targeted by 97 therapeutic drugs (**Figure 5A**; **Supplementary Table 8**). The network included 25 drugs (Cimetidine, Nonoxynol-9, Polyethylene glycol and so on) for Chrna2, 25 drugs (Tetradecyl hydrogen sulfate (ester), Leuprolide, Cianidanol, Methyldopa and so on) for Gnb3, 25 drugs (Ursodeoxycholic acid, Caffeine, Rotavirus vaccine and so on) for Gng7, 25 drugs (Lactitol, Loxapine, Lixisenatide and so on) for Ddit4l, 15 drugs (Hydroxyethyl cellulose, Human adenovirus b serotype 7, Tyrphostin B56 and so on) for Prss55In addition, based on biomarkers, we obtained 73 miRNAs (mmu-miR-6921-5p, mmu-miR-6988-5p, mmu-miR-6998-5p, mmu-miR-7049-3p and so on) and 13 TFs (ETS1, NRF1, USF1 and so on) (**Figure 5B**; **Supplementary Table 9**). Among them, more miRNAs and TFs were predicted by Gng7. Among mmu-miR-7076-5p, mmu-miR-7030-5p, mmu-miR-7075-5p were common targets of Gng7 and Ddit4l, and mmu-miR-505-5p was shared by Gng7 and Chrna2. Besides, TFs SIN3A and MAZ might common to regulate Gng7 and Gnb3. TCF12 was related to Gnb3 and Prss55.

### 3.5 Identification of subtypes based on biomarkers and enrichment analysis

**Figure 6:**
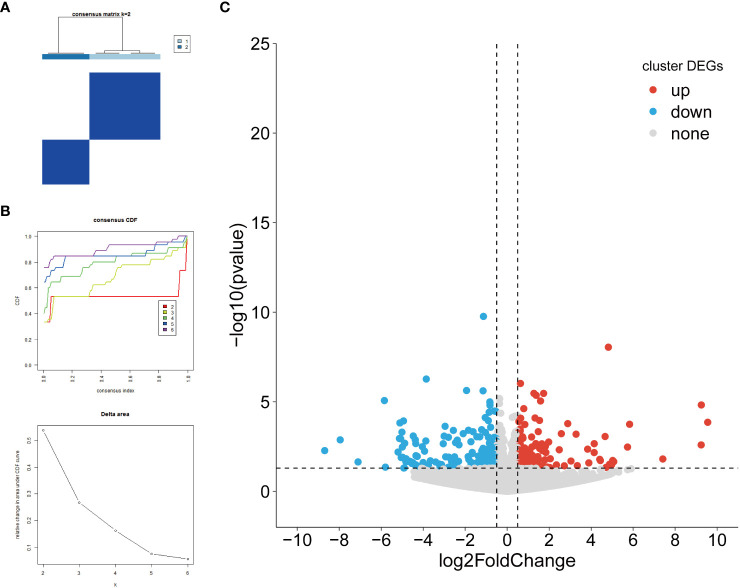
Identification of DEGs among biomarker-based subtypes. **(A)** Heatmap depicts consensus clustering solution (k = 2) for 5 biomarkers in 10 HFpEF samples; **(B)** Delta area curve of consensus clustering indicates the relative change in area under the CDF curve for k = 2 to 6. **(C)** Volcano plot of DEGs between Cluster1 and Cluster2. Red and blue dots indicate upregulated and down-regulated genes, respectively, and gray is the non-significant gene. DEGs, differentially expressed genes; HFpEF, heart failure with preserved ejection fraction; CDF, cumulative distribution function.

In order to perform a comparative analysis of the different subtypes of HFpEF, a consensus clustering analysis based on biomarkers was performed. The consensus clustering results revealed that the samples were clustered into 2 subtypes (Cluster1 and Cluster2), which had the discrimination between subtypes (**Figures 6A, B**). A total of 464 DEGs in different clusters were obtained. Among these clusters, Cluster 1 showed upregulation with 217 genes and downregulation with 247 genes compared to Cluster 2 (**Figure 6C**; **Supplementary Table 10**). The results of functional enrichment analysis indicated that DEGs-clusters were mainly enriched to GO entries such as the regulation of carbohydrate metabolic process and blood circulation, the N-terminal protein amino acid modification, regulatory T cell differentiation and so on (**Supplementary Figure 1**, **Supplementary Table 11**).

### 3.6 PPI network analysis and acquisition of HFpEF-RGs

In order to further explore the crucial genes related to the occurrence and development of HFpEF based on the genes-based clusters of HFpEF, 55 intersected genes (Hmgcs2, Chrna2, Fbp2, Gpam and so on) were acquired by overlapping 971 DEGs (DEGs1) between the HFpEF and control groups and 464 DEGs (DEGs2) in different clusters (**Supplementary Figure 2A**). The PPI network of these 55 genes was built to understand the association among which (**Supplementary Figure 2B**), where Kcnt1 had the most interactions with the remaining genes. Furthermore, five core genes (Kcnt1, Acot1, Kcnc4, Scn3a, and Gpam) in the network were obtained as HFpEF-RGs using MCC algorithm (**Supplementary Figure 2C**).

### 3.7 Immune analysis of biomarkers and HFpEF-RGs

**Figure 7:**
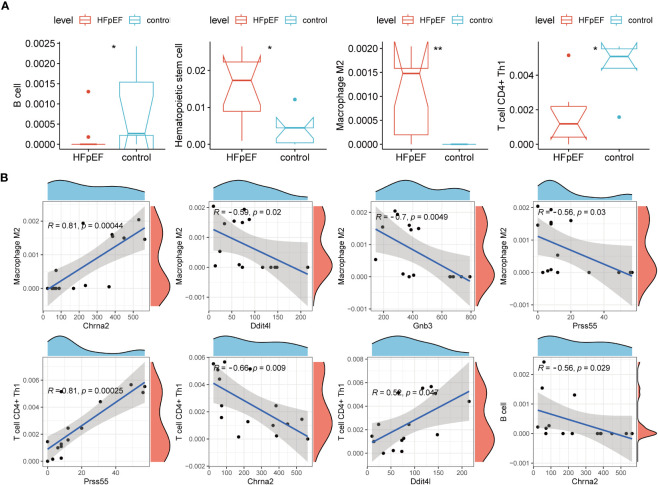
The association of biomarkers with immune microenvironment. **(A)** Immune cell infiltration between two groups by XCELL algorithms and only statistically significant ones are shown. **(B)** Scatter plots show the correlation of biomarkers with the infiltration of Macrophage M2, T cell CD4+ Th1, and B cell. *P < 0.05; **P < 0.01.

To obtain correlations between biomarkers and HFpEF-RGs with immune cells, we performed an immune-related analysis. The Xcell analysis demonstrated significant down-regulation of B cell and T cell CD4+ Th1 in the HFpEF group compared to the control group, whereas Hematopoietic stem cell and Macrophage M2 exhibited higher expression in the HFpEF group (all *P* < 0.05; **Figure 7A**). Additionally, the subsequent correlation analysis (**Figure 7B**) revealed a strong positive correlation between Macrophage M2 and Chrna2 (cor = 0.81, *P* = 0.00044). Furthermore, moderate negative correlations were observed with Gnb3 (cor = -0.7, *P* = 0.0049), Ddit4l (cor = -0.59, *P* = 0.02), and Prss55 (cor = -0.56, *P* = 0.03). As for T cell CD4+ Th1, it demonstrated strong and moderate positive correlations with Prss55 (cor = 0.81, *P* = 0.00025) and Ddit4l (cor = 0.52, *P* = 0.047), respectively, along with a moderate negative correlation with Chrna2 (cor = -0.66, *P* = 0.047). Conversely, B cell only exhibited a significant correlation with Chrna2, indicating a moderate negative relationship (cor = -0.56, *P* = 0.029).

In terms of differential expression between Cluster 1 and Cluster 2, Mast cell and Macrophage were the only cell types that exhibited significant differences, with both being significantly lower in Cluster 1 (**Supplementary Figure 3A**). The correlation analysis results indicated a moderate negative correlation between Acot1 and both Macrophage (cor = -0.54) and Mast cell (cor = -0.53). Furthermore, Macrophage showed a moderate negative correlation with Gpam (cor = -0.7) and Scn3a (cor = -0.53) (all *P* < 0.05; **Supplementary Figure 3B**).

### 3.8 Expression analysis of biomarkers

**Figure 8:**
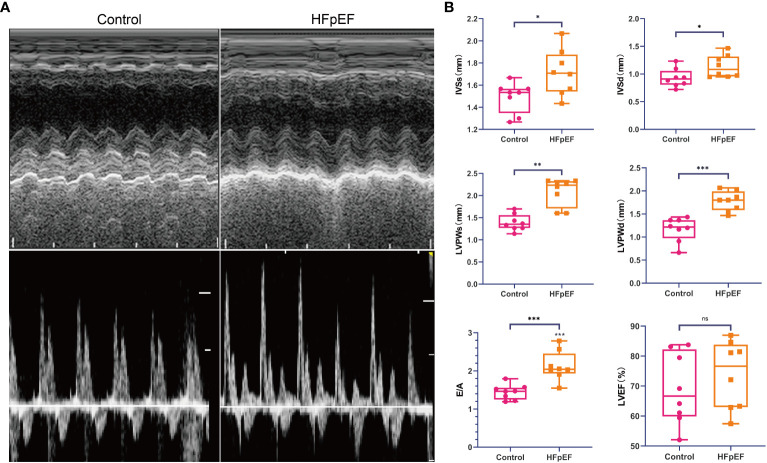
Assessment of cardiac function in the HFpEF mouse model. Echocardiography was performed at 15 weeks after a combination of a HFD (60% kilocalories from fat) and L-NAME (0.5 g l−1 in drinking water) or a standard (chow) diet in mice. **(A)** Representative recordings of echocardiographic images of the LV. **(B)** IVSs, IVSd, LVPWs, LVPWd, E/A, and LVEF were measured by echocardiography. *P < 0.05, **P < 0.01, ***P < 0.001 vs. corresponding control group. ns represents no significance. Data are means ± SEM. HFpEF, heart failure with preserved ejection fraction; HFD, high-fat diet; L-NAME, Nω-nitro-L-arginine methyl ester; LV, left ventricle; IVSs, inter-ventricular septum thickness end systolic; IVSd, inter-ventricular septum thickness end diastolic; LVPWs, left ventricular systolic posterior wall thickness; LVPWd, left ventricular posterior wall thickness end-diastolic; E/A, the early (E) wave peak velocity, representing the passive filling, to the late **(A)** wave peak velocity ratio, representing the active filling due to the atrial contraction; LVEF, left ventricular ejection fraction.

Echocardiography evaluation revealed significant alterations in left ventricular diastolic indexes, including interventricular septal thickness (IVS), left ventricular posterior wall thickness (LVPW), and mitral ratio of peak early to late diastolic filling velocity (E/A), in the HFpEF group compared to normal mice. However, both groups exhibited preserved left ventricular ejection fraction (LVEF) (**Figure 8**).

**Figure 9:**
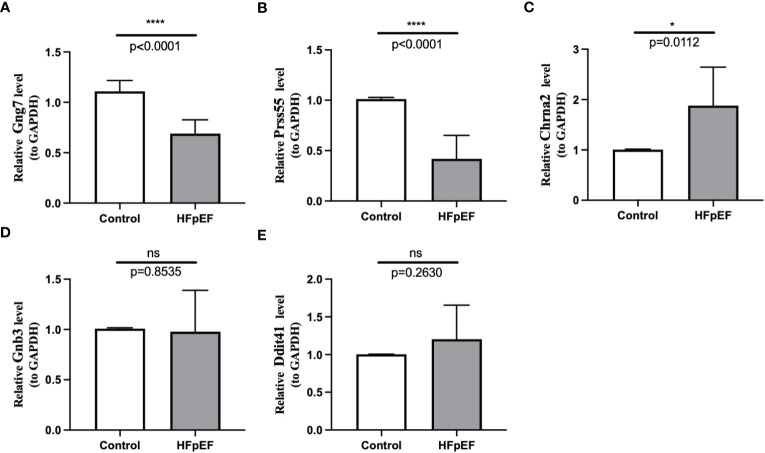
RNA expression of the 5 biomarkers was measured in HFpEF and control samples. RNA expression of Gng7 **(A)**, Prss55 **(B)**, Chrna2 **(C)**, Gnb3 **(D)**, and Ddit4l **(E)** were measured in blood samples using qRT-PCR. P-values were calculated using a two-sided unpaired Student’s t-test. *P < 0.05; ****P < 0.0001; ns represents no significance. HFpEF, heart failure with preserved ejection fraction; qRT-PCR, quantitative reverse transcription polymerase chain reaction.

To further investigate the expression changes of biomarkers in the HFpEF and control groups, eight pairs of HFpEF and control samples were collected and subjected to qRT-PCR. The results demonstrated that the expression levels of Gng7 and Prss55 were significantly lower in HFpEF samples compared to control tissues; conversely, the expression of Chrna2 was higher in HFpEF samples than in control tissues (**Figures 9A–C**), which aligns with findings from public databases. On the other hand, no significant differences in expression were observed for Gnb3 and Ddit4l between the two groups (**Figures 9D, E**).

## 4 Discussion

The pathogenesis of HFpEF remains complex and not fully understood. However, accumulating evidence suggests a significant association between energy metabolism and the development of this disease (20–22). In patients with HFpEF, abnormal energy metabolism in cardiomyocytes leads to pathological changes, including ventricular underfilling, left atrial dilatation, and elevated left atrial pressure (23). Therefore, investigating the expression changes of genes related to energy metabolism in HFpEF can shed light on their roles in disease progression and potentially offer novel targets for the diagnosis and treatment of HFpEF.

After conducting a differential gene analysis and integrating three distinct machine-learning methods in the online HFpEF mouse dataset (GSE180065), cholinergic receptor nicotinic alpha 2 subunit (Chrna2), DNA damage-inducible transcript 4-like (Ddit4l), guanine nucleotide binding protein beta 3 (Gnb3), guanine nucleotide binding protein gamma 7 (Gng7), and serine protease 55 (Pess55) emerged as promising potential diagnostic biomarkers for HFpEF associated with energy metabolism. Subsequent qRT-PCR experiment on the our HFpEF mouse model that were collected from eight HFpEF and eight control samples validated the upregulation of Chrna2, and the downregulations of Gng7 and Prss55 in HFpEF, as did in public database.

Chrna2, the gene encoding acetylcholine receptor subunit α2, is expressed primarily in the nervous system (24). It is involved in signaling of the neurotransmitter acetylcholine and plays an important regulatory role in the nervous system. Additionally, it is closely associated with the onset and progression of a variety of neurological diseases (25). Although no direct association between Chrna2 and cardiovascular disease has been identified, acetylcholine regulates physiological processes in the cardiovascular system. For example, it controls the contraction and diastole of the heart and the dilation and contraction of blood vessels by binding to acetylcholine receptors on the heart and blood vessels (26). Furthermore, the results of Chrna2-GSEA in this study indicate its association with energy metabolism, angiogenesis and development, and cardiac ventricle morphogenesis. These findings suggest that Chrna2, as an energy metabolism-related gene, may have potential diagnostic value for HFpEF.

Ddit4l is a gene that plays an important role in cellular stress and DNA damage response. The expression of Ddit4l is regulated by a variety of factors such as oxygen levels, nutritional status, and cellular stress (27, 28). Bridget Simonson et al.’s study in mice with conditional cardiac-specific overexpression of DDiT4L (29) indicated that in the heart, DDiT4L may be an important pathway for pathological stress (such as metabolic stress) transduction to autophagy through the mTOR signaling pathway. This suggests that DDiT4L may be a therapeutic target in cardiovascular diseases when autophagy and mTOR signaling pathways play important roles. Our study also supports these findings, as the Ddit4l-GSEA results showed a close association between Ddit4l and apoptotic signaling regulation, cardiac conduction, cardiac contraction, and ventricular development. Additionally, through miRNA-Ddit4l network analysis, we identified that miR-669c-5p might regulate Ddit4l. Interestingly, previous research has shown that miR-669c-3p has a protective effect in a mouse model of ischemic stroke by enhancing alternative microglia/macrophage activation and inhibiting MyD88 signaling (30). This evidence further supports the important regulatory role of Ddit4l in the development of heart failure or HFpEF.

Gnb3 is a gene encoding the beta subunit of the G protein. It has been considered a candidate gene for hypertension, autonomic nervous system disorders, and coronary heart disease (31–33). A genetic association study has demonstrated a pathophysiological association between the genetic locus rs5443 (Gnb3) and ventricular remodeling in heart failure (34). In this study, Gnb3-GSEA results consistently showed its involvement in the development of cardiac chambers/ventricles, morphogenesis of the heart/ventricles, and development of ventricular myocardial tissue. These findings suggest that the Gnb3 gene may be associated with structural and functional abnormalities in the hearts of patients with HFpEF. On the other hand, the GNB3 825T allele might be involved in ET-1-induced vasoconstriction in the skin microcirculation (35), and there is association of GNB3 825T variant with increased renal perfusion (36), suggesting the potential relationship of Gnb3 and circulating changes as well as cardiorenal interaction in patients with HFpEF (37). Furthermore, an analysis of the TF-Gnb3 network revealed interesting results about TBP expression. When comparing the right ventricle to the left ventricle or ventricle in healthy controls and HF patients, TBP expression was found to be highly erratic (38). However, in another study conducted on the MI mouse model, TBP expression was stable (39). These findings indicate that the TBP-Gnb3 axis may play a significant role in the pathogenesis of HFpEF.

Gng7 is a gene that encodes the gamma subunit of G protein, which plays a regulatory or translational role in various transmembrane signaling systems (40). Previous studies have shown a correlation between reduced expression of Gng7 and breast cancer (41), lung cancer (42), head and neck cancer (43, 44), and esophageal cancer (45). However, there have been no studies directly linking Gng7 to HFpEF. Our study discovered a potential close association between Gng7 and ventricular development, myocardial tissue development, and cardiac contractile regulation through Gng7-GSEA results. Additionally, our analysis of the miRNA-Gng7 network revealed a close relationship between miR-199a-5p (46), miR-18a-3p (47), miR-491-5p (48), and On the other hand, the GNB3 825T allele might be involved in ET-1-induced vasoconstriction in the skin microcirculation, and there is an association of the GNB3 825T variant with increased renal perfusion, suggesting the potential relationship of Gnb3 with circulating changes, as well as cardiorenal interaction in patients with HFpEF. The predicted interaction of Gng7 and Gnb3 in the BioGRID database might provide more theoretical support to study the underlying mechanism of Gng7 in HFpEF as well (49). These findings suggest that Gng7 may play a role in the pathogenesis of HFpEF.

Prss55 is a gene that encodes a protease known as “proteinase, serine 55”. It belongs to the serine family of proteases and is primarily expressed in testicular tissue (50). Despite being relatively understudied, Prss55 is an important enzyme molecule, and its specific function and mechanism of action remain unknown. Nevertheless, emerging research suggests a potential association between Prss55 and the reproductive system (51, 52). In our study, utilizing Prss55-GSEA, we have identified its potential involvement in crucial biological processes such as angiogenesis and developmental regulation, myocardial tissue development, myocardial contraction, and cardiomyocyte development. To fully comprehend the function and biological significance of Prss55, further investigations are warranted.

In the development of HFpEF, inflammatory response plays a significant role (53). This response is triggered by the infiltration of immune cells, leading to the release of inflammatory mediators in cardiac tissue. The present study’s immunoscape analysis revealed that in the HFpEF group, CD4+ Th1 expression was downregulated in B cells and T cells, while hematopoietic stem cells and M2 macrophages were dominant compared to the control group. B lymphocytes, specialized immune cells present in all jawed vertebrates, have shown increasing association with the heart (54). Although limited, available evidence suggests that B cells may be key players in the development of HFpEF. Biopsies from patients with diastolic dysfunction and controls have shown higher circulating IgG1 and IgG3 levels in patients at higher risk of developing HFpEF (55). Anecdotal evidence points towards the potential improvement of HFpEF with immunomodulation using B-cell-targeted drugs in patients with connective tissue diseases (56). Animal models have also demonstrated the pathogenic role of CD4+ Th1 and Th17 in HF development (57, 58). Sinha et al. found that a higher proportion of CD4+ Th1 cells was associated with a lower risk of developing HF, consistent with the present study (59). Zhang et al. observed increased neutrophil and macrophage infiltration in HFpEF mice hearts, but their results showed an increase in M1 macrophages and a decrease in M2 macrophages, contrasting our findings. However, further studies are needed to explore this discrepancy and provide a rational explanation.

To promote the clinical application of the five diagnostic biomarkers, we used the DrugBank database to predict potential target drugs. Among the predictions, Cianidanol emerged as a co-targeted drug for Chrna2, Gnb3, and Gng7. Cianidanol is a member of the polyphenolic brass subfamily known for its strong antioxidant properties (60). It has been recognized for its potential therapeutic effects in cardiometabolic disorders (61, 62) and cancers (63). Animal and preclinical studies have also demonstrated the vasoprotective effects of cianidanol (64, 65). Additionally, studies suggest that it may offer a unique approach to reducing atherosclerosis (66). Esmolol, which is currently used in the treatment of cardiovascular disease, has been predicted as a co-targeted agent targeting Chrna2 and Gnb3. This cardioselective β-blocker has shown effectiveness in controlling tachycardia and acute ischemic elevated hemodynamic parameters in patients with heart disease (67). Perindopril, a co-target of Chrna2 and Gng7, is an angiotensin-converting enzyme inhibitor indicated for the treatment of hypertension (68). Furthermore, a review has shown that Perindopril, when combined with other antihypertensives, minimizes cardiovascular events (69). Methyldopa has been identified as a co-targeted agent of Ddit4l and Gng3. This drug has been used in the treatment of hypertension since the 1960s (70). Lovastatin, on the other hand, is a co-targeted drug for Ddit4l and Gng7 and is commonly used to treat coronary heart disease and hypercholesterolemia (71). Additionally, other potentially targeted drugs such as Rosuvastatin (72), Mexiletine (73), Labetalol (74), Lomitapide (75), and Metolazone (76) may be beneficial in the treatment of cardiovascular disease. However, no reports or studies have been conducted on the association of these targeted agents with HFpEF or with Chrna2, Ddit4l, Gnb3, and Gbg7. Therefore, further studies are needed to confirm their potential mechanisms of action. As for Prss55, DrugBank analysis showed that there are no potential targeted drugs directly related to cardiovascular disease treatment. This finding deserves further attention and investigation.

There are some shortcomings of this study that need to be noted. First, the study was based on bioinformatic analysis of HFpEF mouse transcriptome data from the GEO database. However, the sample size was relatively small, which may limit the statistical reliability and generalizability of the results. Second, the study identified five diagnostic biomarkers associated with energy metabolism, but expression validation in human clinical samples was not performed. It is crucial to perform clinical sample validation to determine the practical application and validity of these markers in patients with HFpEF. Although PCR validation was conducted using HFpEF mice, the results did not successfully validate the differential expression of Gnb3 and Ddit4l between the control and HFpEF groups. This lack of validation may be attributed to individual differences between samples, the variability of experimental techniques, and the complexity of gene regulation. Thirdly, although a consistent cluster analysis of HFpEF was performed based on five diagnostic biomarkers associated with energy metabolism, practical clinical significance could not be assigned to these subclasses due to the current lack of human data. Additionally, bioinformatics analysis, while important in HFpEF research, still suffers from limitations such as the quality and consistency of data, data processing, and choice of analysis methods. Furthermore, this study used a mouse model to study HFpEF, but the mouse model cannot fully reflect the complex pathological process and biological characteristics of human HFpEF, while enough public data on human HFpEF samples could not be found for analysis. Therefore, caution should be exercised when applying these findings to clinical practice. To overcome these shortcomings, future studies should aim to increase the sample size, perform human clinical sample validation, and validate and confirm the clinical application potential of these analytical results by integrating other experimental models and methods.

## 5 Conclusion

The analysis of HFpEF mouse transcriptome data from the GEO database successfully identified five diagnostic biomarkers, namely Chrna2, Ddit4l, Gnb3, Gng7, and Prss55, which are associated with energy metabolism. These biomarkers demonstrated a strong ability to distinguish between HFpEF samples and control samples. Furthermore, the GSEA analysis revealed their potential involvement in crucial biological processes like ventricular development and cardiac contraction. These findings have significant implications for understanding the pathogenesis of HFpEF and identifying potential disease biomarkers. Moreover, these biomarkers hold promise as early diagnostic and predictive indicators for HFpEF, offering new prospects for personalized therapy.

## Data availability statement

The original contributions presented in the study are included in the article/**Supplementary Material**. Further inquiries can be directed to the corresponding author.

## Ethics statement

The animal study was approved by The Ethics Committee of Shaanxi Provincial People’s Hospital, Xi’an, Shaanxi, China. The study was conducted in accordance with the local legislation and institutional requirements.

## Author contributions

QG: Conceptualization, Data curation, Formal analysis, Investigation, Methodology, Project administration, Resources, Software, Supervision, Validation, Visualization, Writing – original draft, Writing – review and editing. QZ: Conceptualization, Data curation, Formal analysis, Investigation, Methodology, Project administration, Resources, Software, Supervision, Validation, Visualization, Writing – original draft, Writing – review and editing. MD: Conceptualization, Data curation, Formal analysis, Investigation, Methodology, Project administration, Resources, Supervision, Validation, Writing – original draft, Writing – review and editing. LL: Conceptualization, Data curation, Formal analysis, Investigation, Methodology, Project administration, Resources, Supervision, Validation, Visualization, Writing – original draft, Writing – review and editing. HY: Conceptualization, Data curation, Formal analysis, Investigation, Methodology, Project administration, Resources, Software, Supervision, Validation, Visualization, Writing – original draft, Writing – review and editing.

## Supplementary material

Supplementary Figure 1 GO functional enrichment analysis of 464 DEGs in Cluster1 and Cluster2 samples. **(A)** Relationship among the top 10 enriched BP terms and targets is represented in a chord plot. **(B)** Relationship among the top 10 enriched CC terms and targets is represented in a chord plot. **(C)** Relationship among the top 10 enriched mf terms and targets is represented in a chord plot. The colors of the nodes range from red to blue in descending order of logFC values. The genes are ordered according to logFC values. GO: Gene Ontology; DEGs: differentially expressed genes; BP: biological process; CC: cellular component; MF: molecular function.

Supplementary Figure 2 Construction of PPI network and identification of hub genes. **(A)** The Venn diagram of DEGs1 and DEGs2. DEGs1 represents DEGs between control and HFpEF groups, and DEGs2 represents DEGs between cluster 1 and cluster 2. **(B)** PPI network of the DEGs between cluster 1 and cluster 2. The PPI network of DEGs was constructed using Cytoscape. **(C)** The top 5 key genes were screened through the PPI network map. PPI: protein-protein interaction; DEGs: differentially expressed genes.

Supplementary Figure 3 The association of hub genes with immune microenvironment. **(A)** Immune cell infiltration between two cluster s by XCELL algorithms and only statistically significant ones are shown. **(B)** Scatter plots show the correlation of hub genes with the infiltration of Mast cell and Macrophage. **P* < 0.05.. Primer sequences of PCR.

Supplementary Table 1 DEGs between the HFpEF and control groups in the GSE180065 dataset.

Supplementary Table 2 Expression of identified DE-EMRGs in the GSE180065 dataset.

Supplementary Table 3 GSEA-GO analysis results for Chrna2.

Supplementary Table 4 GSEA-GO analysis results for Gng7.

Supplementary Table 5 GSEA-GO analysis results for Ddit4l.

Supplementary Table 6 GSEA-GO analysis results for Prss55.

Supplementary Table 7 GSEA-GO analysis results for Gnb3.

Supplementary Table 8 Potential target drugs for 5 biomarkers predicted by DrugBank.

Supplementary Table 9 NetworkAnalyst predicted miRNA-biomarker and biomarker-TF relationship pairs.

Supplementary Table 10 DEGs between the Cluster1 and Cluster2.

Supplementary Table 11 Results of GO analysis of DEGs-clusters.
