# Supplementary figures and images for: Diagnostic potential of energy metabolism-related genes in heart failure with preserved ejection fraction
Source: Front Endocrinol (Lausanne). 2023 Nov 27;14:1296547. doi: 10.3389/fendo.2023.1296547 (PMC10711684; doi:10.3389/fendo.2023.1296547)

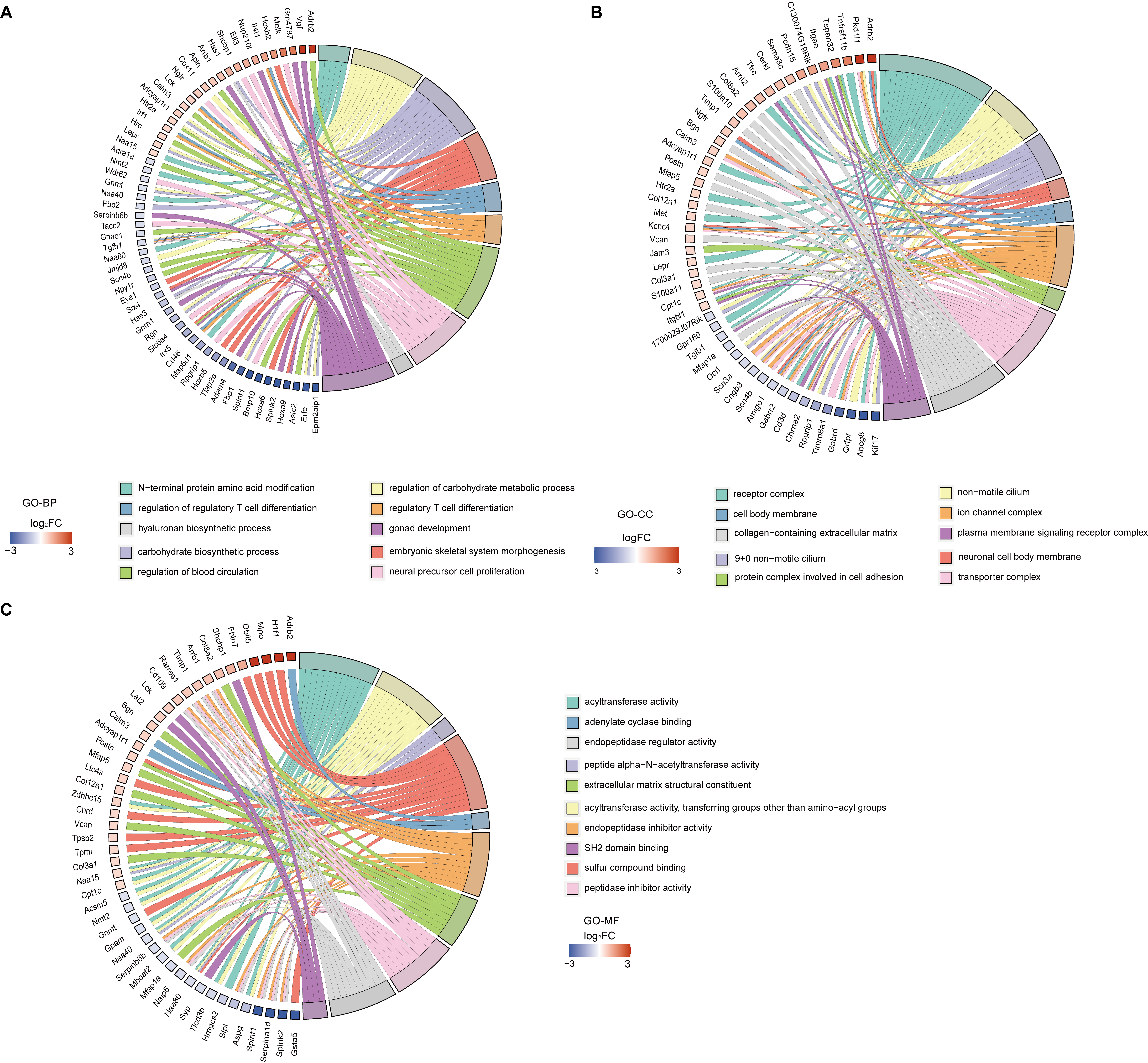

Supplement: Supplementary Figure 1 — GO functional enrichment analysis of 464 DEGs in Cluster1 and Cluster2 samples. (A) Relationship among the top 10 enriched BP terms and targets is represented in a chord plot. (B) Relationship among the top 10 enriched CC terms and targets is represented in a chord plot. (C) Relationship among the top 10 enriched mf terms and targets is represented in a chord plot. The colors of the nodes range from red to blue in descending order of logFC values. The genes are ordered according to logFC values. GO: Gene Ontology; DEGs: differentially expressed genes; BP: biological process; CC: cellular component; MF: molecular function. [file Image_1.tif]

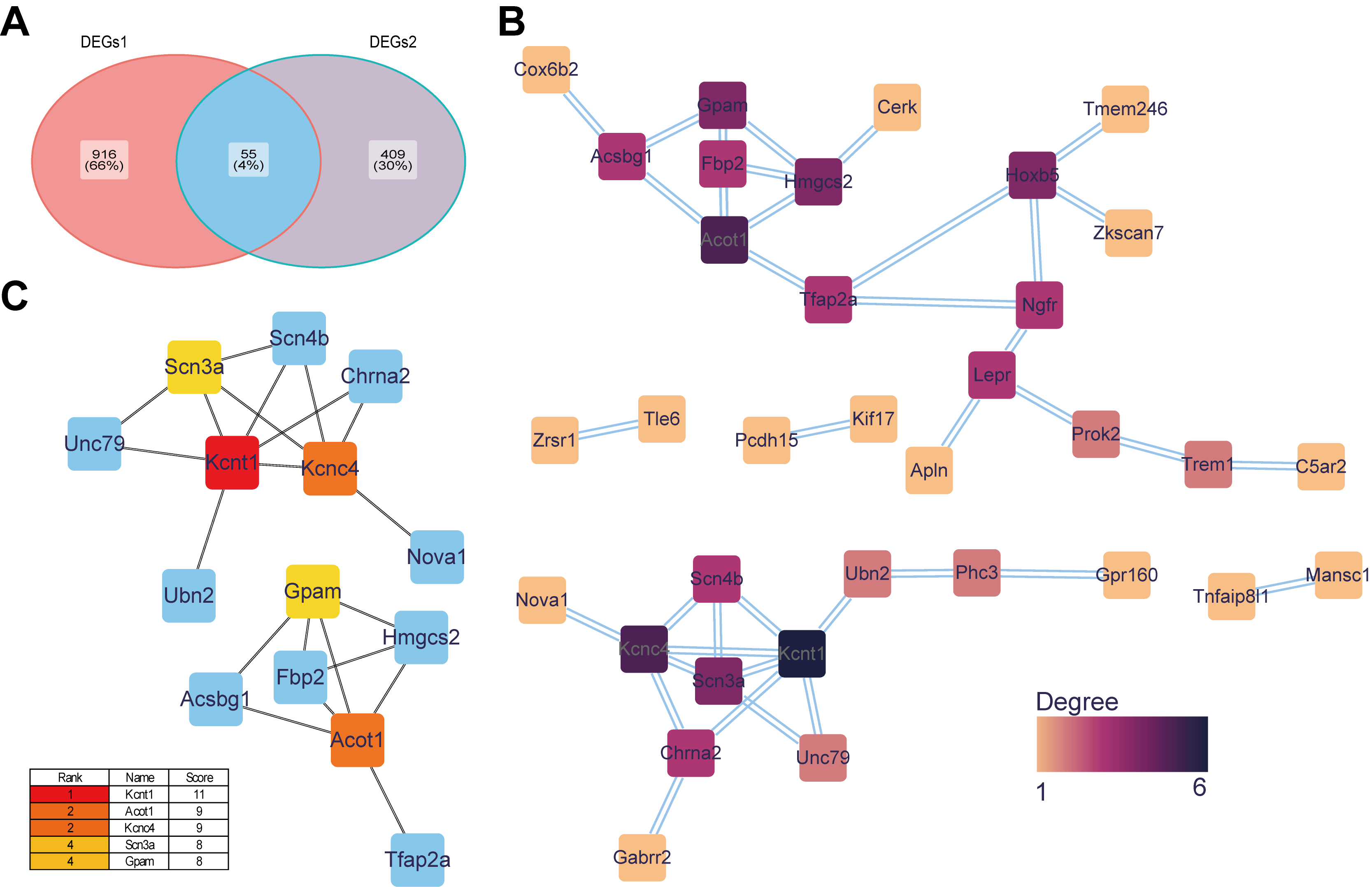

Supplement: Supplementary Figure 2 — Construction of PPI network and identification of hub genes. (A) The Venn diagram of DEGs1 and DEGs2. DEGs1 represents DEGs between control and HFpEF groups, and DEGs2 represents DEGs between cluster 1 and cluster 2. (B) PPI network of the DEGs between cluster 1 and cluster 2. The PPI network of DEGs was constructed using Cytoscape. (C) The top 5 key genes were screened through the PPI network map. PPI: protein-protein interaction; DEGs: differentially expressed genes. [file Image_2.tif]

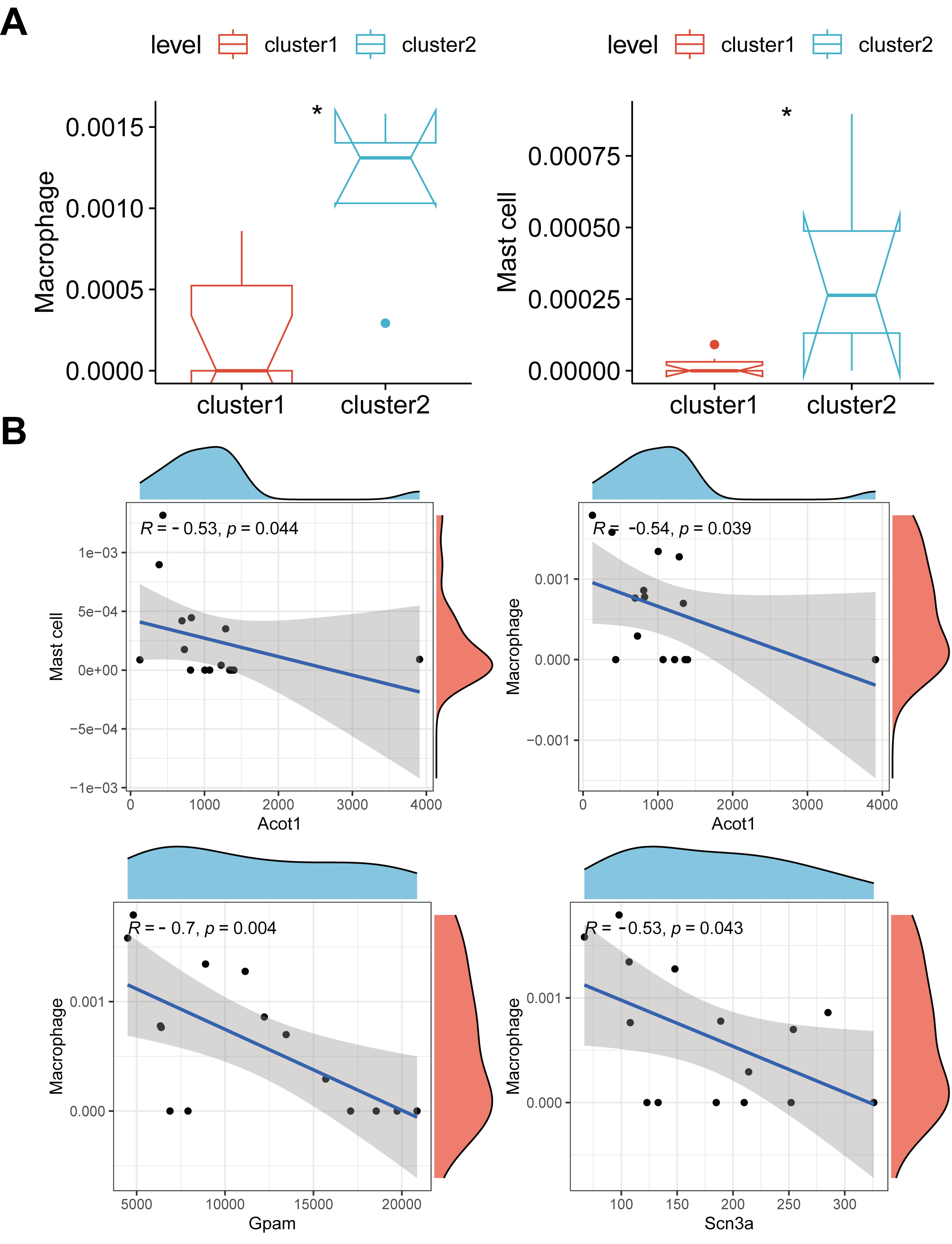

Supplement: Supplementary Figure 3 — The association of hub genes with immune microenvironment. (A) Immune cell infiltration between two cluster s by XCELL algorithms and only statistically significant ones are shown. (B) Scatter plots show the correlation of hub genes with the infiltration of Mast cell and Macrophage. *P < 0.05.. Primer sequences of PCR. [file Image_3.tif]
